# The Development of a Novel Therapeutic Strategy to Target Hyaluronan in the Extracellular Matrix of Pancreatic Ductal Adenocarcinoma

**DOI:** 10.3390/ijms18030600

**Published:** 2017-03-09

**Authors:** Daisuke Kudo, Akiko Suto, Kenichi Hakamada

**Affiliations:** Gastroenterological Surgery, Hirosaki University Graduate School of Medicine, 5 Zaifu-cho, Hirosaki, Aomori 036-8562, Japan; aikopapa@hirosaki-u.ac.jp (D.K.); underthebridge_trol@yahoo.co.jp (A.S.)

**Keywords:** hyaluronan, 4-methylumbelliferone, pancreatic ductal adenocarcinoma, extracellular matrices

## Abstract

Pancreatic ductal adenocarcinoma (PDAC) is one of the most lethal diseases to affect humans, regardless of whether patients receive multimodal therapy (including surgery, radiotherapy, and chemotherapy). This resistance to intervention is currently considered to be caused by the desmoplastic change of the extracellular matrix (ECM) in PDAC tissues, which is characterized by the accumulation of cancer-associated fibroblasts, collagen, proteoglycan, and hyaluronan. Among these ECM components, hyaluronan has attracted interest because various studies have indicated that hyaluronan-rich PDAC is correlated with the progressive properties of cancer cells, both in experimental and clinical settings. Hence, the reduction of hyaluronan in cancer tissue may represent a novel therapeutic approach for PDAC. 4-methylumbelliferone (4-MU) is a derivative of coumarin that was reported to suppress the synthesis of hyaluronan in cultured human skin fibroblasts in 1995. As an additional study, our group firstly reported that 4-MU reduced the hyaluronan synthesis of mouse melanoma cells and exerted anti-cancer activity. Subsequently, we have showed that 4-MU inhibited liver metastasis in mice inoculated with human pancreatic cancer cells. Thereafter, 4-MU has been accepted as an effective agent for hyaluronan research and is expected to have clinical applications. This review provides an overview of the interaction between PDAC and hyaluronan, the properties of 4-MU as a suppressor of the synthesis of hyaluronan, and the perspectives of PDAC treatment targeting hyaluronan.

## 1. Introduction

Pancreatic ductal adenocarcinoma (PDAC) is now the fourth cause of cancer-related deaths in the US. The data show that the 5-year survival rate of patients with PDAC after the initiation of intervention is <10%, which is the worst of all malignancies [1]. Three factors are likely to be responsible for this poor prognosis. Firstly, patients with PDAC have no specific symptoms; thus, the disease may reach a highly advanced stage before detection. Actually, 85% of PDAC patients have inoperable locally advanced cancer and/or distant metastasis at the initial diagnosis [2]. Secondly, even if they undergo surgical resection, a potentially curative treatment, PDAC can be expected to relapse after a relatively short period because invisible micrometastasis has already occurred before surgery [3]. Thirdly, PDAC displays considerable resistance to chemotherapy. The administration of intensive chemotherapy can prolong the survival time by 2–6 months [4,5,6,7]. These difficulties in PDAC treatment are partly due to the desmoplastic change of the extracellular matrix (ECM), which is characterized by the accumulation of cancer-associated fibroblasts (CAF) and increased fibrous ECM components, including collagen, proteoglycans, and hyaluronan [8]. This remodeling of the microenvironment surrounding the cancer cells contributes to the elevation of the internal pressure in the cancerous tissue [9]. Moreover, in this process, the deposition of hyaluronan causes the compression of the intratumoral microvasculature and consequently prevents the drug from reaching the cancer cells [10]. In addition, hyaluronan works as a ligand for some receptors on the cell membrane and enhances the progression of cancer. Thus, the control of hyaluronan is a considered to be a promising approach in efforts to treat this irremediable disease.

## 2. Hyaluronan and Its Role in Progression of Pancreatic Ductal Adenocarcinoma

Hyaluronan was firstly described as hyaluronic acid, which was extracted from the vitreous of bovine eyes in 1934 [11]. Its chemical structure was revealed to be non-sulfated linear glycosaminoglycan composed of repeated β-1,4-d-glucuronic acid-β-1,3-d-*N*-acetylglucosamine disaccharide unit [12]. The synthesis of hyaluronan on the plasma membrane involves three different hyaluronan synthase (HAS) proteins [13]; these lengthen hyaluronan in the ECM to a size as large as 10^5^–10^6^ Da [14]. Hyaluronan has the ability to retain a large amount of water molecules and to create viscous gels when combined with other ECM components such as glycosaminoglycans, proteoglycans, and collagens [15]. Hyaluronan ubiquitously exists in the ECM of the vast majority of organs and provides a scaffold for intercellular signal transduction. Furthermore, it plays an important role in pathophysiological processes, including embryogenesis [16], proliferation [17], inflammation [18], wound healing [19], and carcinogenesis. Several studies have reported that the excessive accumulation of hyaluronan in cancer tissues is associated with the worse prognosis after surgery in breast [20], gastric [21], and colorectal [22] cancer. In the case of pancreatic adenocarcinoma, the quantitative analysis of the hyaluronan content in resected cancerous tissue revealed that the level was 4–12 times that in normal pancreatic tissues [23,24,25]. This also has a clinical impact on the poor overall survival rate after surgery.

In cancer progression, hyaluronan acts as a principle ligand, binding to receptors including CD44 and RHAMM [26,27,28]. These activate the PI3K/Akt and ERK1/2 signaling pathways and result in proliferation, angiogenesis, cytoskeleton rearrangement, and invasion [29,30]. In addition, CD44 has been recognized as a cancer stem cell marker of PDAC and is involved in both multidrug resistance and epithelial mesenchymal transition, which protects cancer cells from chemotherapeutic agents [31,32]. The intercellular signal transduction between the cancer cells and the cancer-associated fibroblasts promotes secretion of hyaluronan into the extracellular matrices of PDAC. They produce hormones, growth factors and cytokines to produce a large amount of hyaluronan that stimulates their malignant cycles. As a result, the cancer cells acquire an appropriate microenvironment to survive, proliferate, and invade by the interaction between hyaluronan and its receptors. The other important role of the accumulation of hyaluronan in cancer progression is that it increases the interstitial fluid pressure (IFP), which makes drug perfusion difficult [9,10]. Hyaluronidase treatment was shown to reduce the IFP within two hours in a mouse model of PDAC and to significantly improve the response to gemcitabine, one of the key drugs for PDAC treatment [33]. Hence, reducing the level of hyaluronan in cancer tissue is expected to be an effective approach to treating PDAC.

## 3. The Discovery of 4-Methylumbelliferone as an Inhibitor of Hyaluronan Synthesis and Its Mechanism of Action

The amount of hyaluronan in the ECM is regulated by three synthases (HAS1, HAS2, and HAS3 [34]) and six isoforms of hyaluronidase (HYAL1, HYAL2, HYAL3, HYAL4, PHYAL4, and PH20 [35]). Their genetic manipulation has revealed the importance of hyaluronan in the progression of cancer. However, the expression of these enzymes in pancreatic cancer cells is heterogeneous [36]. This suggests that the development of antibodies for HAS or promotors for each hyaluronidase may not result in a valid response in most patients with PDAC. Thus, many studies have sought compounds that reduce the synthesis of hyaluronan (Table 1) [37,38,39,40,41,42,43,44,45,46,47,48,49,50,51,52,53,54]. Among them, 4-methylumbelliferone (4-MU), which stably suppresses the synthesis of hyaluronan in vitro and in vivo, has been widely accepted because of its mechanism of action and because it is harmless to organisms.

**Figure 1:**
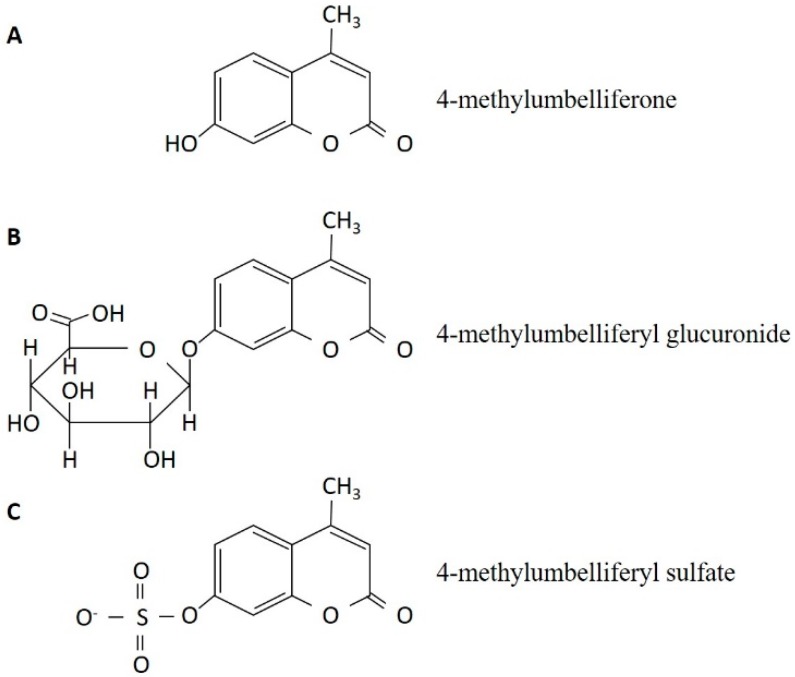
The chemical structure of 4-methylumbelliferone (**A**) and its metabolites, 4-methylumbelliferyl glucuronide (**B**) and 4-methylumbelliferyl sulfate (**C**).

4-MU is one of the coumarin compounds; hydroxylation is observed in position seven of coumarin, while methylation is observed in position four. Thus, its IUPAC name is 7-hydroxy-4-methylcoumarin (Figure 1A). 4-MU and its conjugates, including 4-methylumbelliferyl glucuronide (4-MUG, Figure 1B) and 4-methylumbelliferyl sulfate (4-MUS, Figure 1C), have been used as markers of enzymatic activity because 4-MU has fluorescence activity (excitation wave-length 380 nm and emission wave-length 454 nm) [55]. During an experiment that involved the synthesis of 4-methlumbelliferyl xyloside, Nakamura et al. firstly reported (in 1995) that 4-MU inhibited the synthesis of hyaluronan by cultured human skin fibroblast [48]. Thereafter, our group reported that the suppression of hyaluronan synthesis had anti-cancer effects in cultured mouse melanoma cells, [56], and the administration of 4-MU to C57BL/6J mice inoculated with the melanoma cells reduced tumor size and distant metastases [57]. Subsequently, we have shown that 4-MU reduced liver metastasis of KP1-NL (a human pancreatic cancer cell line) in vivo and demonstrated that its effect was enhanced by the co-administration of gemcitabine [58]. Over time, 4-MU has been come to be used as an effective and specific suppressor of hyaluronan synthesis in various malignancies, including prostate cancer [59], esophageal cancer [60], colorectal cancer [61], breast cancer [62], liver cancer [63], osteosarcoma [64], and leukemia [65].

Two possible molecular mechanisms of 4-MU have been proposed: the depletion of intracellular UDP-glucuronic acid (an HAS substrate) [66] and the downregulation of the HAS mRNA level [67]. Among these mechanisms, the former is mainly accepted because 4-MU is metabolized to 4-methylumbelliferyl glucuronide (Figure 1B) by UDP-glucuronic acid transferase, and consequently decreases the source of hyaluronan through the consumption of the cellular UDP-glucuronic acid pool. Jokela et al. reported that mannose inhibited hyaluronan synthesis through UDP-*N*-acetyl-hexosamines (another HAS substrate) depletion in cultured rat epidermal keratinocytes [51]. They showed 4-MU reduced the cellular UDP-glucuronic acid pool and its effect was not enhanced by the addition of mannose. These data fit the fact that hyaluronan is composed of glucuronic acid and *N*-acetyl-glucosamine by 1:1. Another theory about HAS expression is still controversial, because some studies have reported that 4-MU upregulated the HAS mRNA level in the human osteosarcoma cells [64] and human pancreatic ductal adenocarcinoma cells [68]. They described that upregulation of HAS was caused by the positive feedback as a result of hyaluronan depletion. Therefore, further study to reveal the effect of 4-MU on HAS gene, protein and its pathway would be necessary. The mechanisms, which are independent of any specific receptor, allow 4-MU to inhibit the synthesis of hyaluronan—irrespective of species, organ and cell type—because the structure of hyaluronan is homologous in all types of eukaryotes. Therefore, other substrates such as hormones and growth factors (Table 1), which enhance the HAS gene promotor, have not been employed as a specific inhibitor of hyaluronan synthesis. Another reason why 4-MU is frequently employed in hyaluronan research, especially in vivo, is its harmless nature. Indeed, the LD_50_ of 4-MU is reported to be 3150 mg/kg when it was orally administered to mice in an acute toxicity test. In a chronic toxicity test, animals were able to tolerate a daily dose of 1000 mg/kg for 26 weeks (described in Japanese). These tests were performed to investigate the safety of “hymecromone” (the drug name of 4-MU), which has been used as a choleretic and anti-spasmodic agent for humans in many countries [69,70]. The background information that is already available from human studies is an advantage of 4-MU with regard to its clinical application; however, it is not currently approved for use in either the US. or Japan, and a clinical trial with the approval of each government is therefore necessary.

## 4. The Alteration of the Extracellular Matrix in Pancreatic Ductal Adenocarcinoma through Reduction of Hyaluronan

The development of novel cancer treatments that target hyaluronan by altering the ECM represents a pioneering approach to the treatment of PDAC. Recently, a phase Ib study investigated the administration of PEGylated recombinant human PH20 with gemcitabine in patients with PDAC [71]. The results indicated its efficacy, especially in the case of tumoral hyaluronan rich disease. In the preclinical study, the intravenous administration of PEGPH20 to tumor-inoculated mice reduced the level of hyaluronan and the interstitial fluid pressure in the nodule, and enhanced the activity of both docetaxel and liposomal doxorubicin [72]. This series of studies shows that a reduction of the level of hyaluronan in PDAC tissue allows anticancer agents to reach the cancer cells. This strategy is similar to the strategy of 4-MU treatment. In fact, we previously reported that the combination of 4-MU and gemcitabine enhanced the anticancer activity of gemcitabine [58]. We showed that pancreatic cancer cells are each surrounded by hyaluronan-rich matrices, and that the addition of 4-MU into the medium inhibited the formation of the envelope, which promoted the perfusion of gemcitabine. In another human pancreatic cancer cell-bearing mouse model, electron microscopic observation revealed that 4-MU decreased the amount of hyaluronan in tumors on the backs of animals and altered the intercellular space, causing it to become coarse [73]. Furthermore, we reported that 4-MU increased the survival rate of mice that were intraperitoneally inoculated with human PDAC cells, and that the intratumoral level of hyaluronan was reduced by 30% in comparison to control mice [68]. These results indicate that orally administered 4-MU is distributed throughout the body and that it will exert anticancer activities against both primary and distant metastatic disease. In addition, reduction of pericellular hyaluronan by 4-MU causes suppression of proliferation, migration and invasion activities. These anticancer properties were also observed when anti-CD44 antibody was administered into the culture medium, but 4-MU affects neither the expression of CD44 protein on the cellular membrane nor hyaluronan binding to the receptor. The result indicates that 4-MU exerts an anticancer property via suppression of hyaluronan synthesis, and subsequently reduces the interaction between hyaluronan and CD44. The addition of exogenous high molecular hyaluronan into the culture medium counteracts the inhibition of the cellular migration and invasion by 4-MU; on the other hand, the cellular proliferation is not recovered by the exogenous high molecular hyaluronan. This suggests that the cancer cells require different lengths of hyaluronan molecules to survive, copy and locomotion. The size of hyaluronan in the extracellular matrices is principally controlled by the hyaluronidases, but the influence of 4-MU on these enzymes has not been studied. Because Nakamura et al have reported that 4-MU did not alter the chain length of hyaluronan produced by human skin fibroblasts [48], it would not affect the hyaluronidase activity and expression. The injection of PEGylated recombinant human PH20, a kind of hyaluronidase, showed measurable success in the treatment of PDAC patients. The principal object of this treatment is the reduction of interstitial fluid pressure in the PDAC tissue by the degradation of hyaluronan, and 4-MU would also produce a similar effect by inhibition of hyakuronan synthesis. Moreover, 4-MU can be administered repetitively to animals without adverse effects; consequently, it would permanently provide hyaluronan knockdown extracellular matrices during administration.

**Figure 2:**
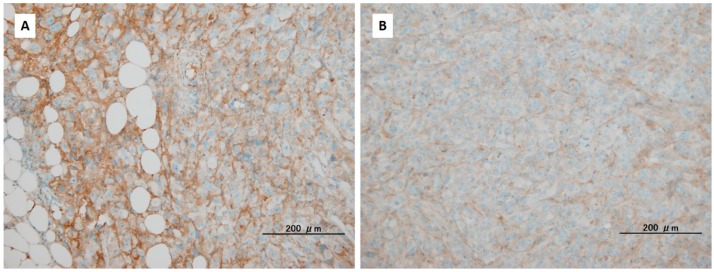
The reduction of hyaluronan accumulation in cancer tissues of mice inoculated with human pancreatic ductal adenocarcinoma cells by 4-methylumbelliferone (4-MU). The animals were treated without 4-MU (**A**) or with 4-MU (**B**). Hyaluronan in the pancreatic tumors was detected using immunohistochemical staining with hyaluronan-binding proteins. Black bars represent 200 mm.

The combination of hyaluronan knockdown and immunotherapy is another direction that may offer hope in efforts to conquer PDAC. As described above, the reduction of hyaluronan in the tumor tissue reduces the IFP and promotes drug perfusion via the release of compressed microvessels. Several studies have reported that an immunological approach, such as the administration of peptides [74], tumor-specific cytotoxic T cells [75], and immune checkpoint inhibitors [76], showed efficacy in PDAC treatment. The alteration of the cancer microenvironment by the control of hyaluronan may also increase the sensitivity of these immune targeting agents (Figure 2). The accumulation of tumor infiltrating T cells in surgically resected PDAC is correlated with better postoperative survival [77,78], and the current development of iPS technology will enable large amounts of cytotoxic lymphocytes with cancer-specific antigens to be obtained in each patient [79]. Thus, the control of hyaluronan will become an adjuvant treatment that can be used in combination with these immunotherapies, which will enhance their efficacy through the remodeling of the ECM of cancer cells.

## 5. Conclusions

Hyaluronan has a simple chemical structure that is homologous in both normal and cancer tissues. Thus, the complete knockdown or excessive reduction of hyaluronan has lethal effects in the host or organ. Indeed, PDAC patients who were treated with PEGPH20 had several adverse events, including musculoskeletal pain, peripheral edema, and thromboembolic events [71]. Thus, achieving a moderate reduction in the hyaluronan level will be important for its clinical application in combination with chemotherapy and/or immunotherapy. Some studies have reported that the excessive accumulation of hyaluronan in inflammatory tissues leads to organ dysfunction, and 4-MU suppressed destruction of the tissues [80,81,82]. From this viewpoint, further studies of 4-MU are considered necessary.

## Figures and Tables

**Table 1:** The inhibitors of hyaluronan synthesis.

No.	Substance	Cells	Author (year)	Ref.
1.	anti-inflammatory steroids	Human skin fibroblasts	Saarni, H. et al. (1978)	[37]
2.	Monensin	Rat fibrosarcoma cells	Goldberg, R.L. and Toole, B.P. (1983)	[38]
3.	Cyclofenil diphemol	Rat chondrocytes	Mason, R.M. et al. (1984)	[39]
4.	Periodae-oxidized UDP-GlcNAc	Human fibrosarcoma cells	Prehm P. (1985)	[40]
5.	n-Butylate	Rat liver fat-storing cells	Gressner, A.M. and Haarmann, R. (1988)	[41]
6.	Dexamethasone	Human skin fibroblasts	Smith, T.J. (1988)	[42]
7.	All-trans retinoic acid	Human skin fibroblasts	Smith, T.J. (1990)	[43]
8.	*p*-Nitrophenol-β-d-xyloside	Rat liver fat-storing cells	Gressner, A.M. (1991)	[44]
9.	Genistein	Rabbit mesothelial cells	Honda, A. et al. (1991)	[45]
10.	Suramin	Mouse skin fibroblasts	August, E.M. et al. (1993)	[46]
11.	Vanadate	Mouse skin fibroblasts	Zaharevitz, D.W. et al. (1993)	[47]
12.	4-methylumbelliferone	Human skin fibroblasts	Nakamura, T. et al. (1995)	[48]
13.	Fluoxetine, amitriptyline	Human synovial cells	Yaron, I. et al. (1999)	[49]
14.	Vesnarinone	Human myofibroblasts	Ueki, N. et al. (2000)	[50]
15.	Mannose	Human myofibroblasts	Jokela, T.A. et al. (2008)	[51]
16.	Methyl-β-cyclodextrin	Human breast cancer cells	Kultti, A. et al. (2010)	[52]
17.	Estradiol	Human vascular smooth muscle cells	Freudenberger, T. et al (2011)	[53]
18.	Collagen fragments	Human skin fibroblasts	Röck, K. et al. (2011)	[54]

## References

[1] Siegel R.L., Miller K.D., Jemal A. (2016). Cancer statistics, 2016. CA Cancer J. Clin..

[2] Stathis A., Moore M.J. (2010). Advanced pancreatic carcinoma: Current treatment and future challenges. Nat. Rev. Clin. Oncol..

[3] Lombardi L., Troiano M., Silvestris N., Nanni L., Latiano T.P., Di Maggio G., Cinieri S., Di Sebastiano P., Colucci G., Maiello E. (2012). Combined modality treatments in pancreatic cancer. Expert Opin. Ther. Targets.

[4] Burris H.A., Moore M.J., Andersen J., Green M.R., Rothenberg M.L., Modiano M.R., Cripps M.C., Portenoy R.K., Storniolo A.M., Tarassoff P. (1997). Improvements in survival and clinical benefit with gemcitabine as first-line therapy for patients with advanced pancreas cancer: A randomized trial. J. Clin. Oncol..

[5] Moore M.J., Goldstein D., Hamm J., Figer A., Hecht J.R., Gallinger S., Au H.J., Murawa P., Walde D., Wolff R.A. (2007). Erlotinib plus gemcitabine compared with gemcitabine alone in patients with advanced pancreatic cancer: A phase III trial of the National Cancer Institute of Canada Clinical Trials Group. J. Clin. Oncol..

[6] Conroy T., Desseigne F., Ychou M., Bouché O., Guimbaud R., Bécouarn Y., Adenis A., Raoul J.L., Gourgou-Bourgade S., de la Fouchardière C. (2011). FOLFIRINOX versus gemcitabine for metastatic pancreatic cancer. N. Engl. J. Med..

[7] Von Hoff D.D., Ervin T., Arena F.P., Chiorean E.G., Infante J., Moore M., Seay T., Tjulandin S.A., Ma W.W., Saleh M.N. (2013). Increased survival in pancreatic cancer with nab-paclitaxel plus gemcitabine. N. Engl. J. Med..

[8] Liu H., Ma Q., Xu Q., Lei J., Li X., Wang Z., Wu E. (2012). Therapeutic potential of perineural invasion, hypoxia and desmoplasia in pancreatic cancer. Curr. Pharm. Des..

[9] DuFort C.C., DelGiorno K.E., Hingorani S.R. (2016). Mounting Pressure in the Microenvironment: Fluids, Solids, and Cells in Pancreatic Ductal Adenocarcinoma. Gastroenterology.

[10] DuFort C.C., DelGiorno K.E., Carlson M.A., Osgood R.J., Zhao C., Huang Z., Thompson C.B., Connor R.J., Thanos C.D., Scott Brockenbrough J. (2016). Interstitial pressure in pancreatic ductal adenocarcinoma is dominated by a gel-fluid phase. Biophys. J..

[11] Mayer K., Palmer J.W. (1934). The polysaccharide of the vitreous humor. J. Biol. Chem..

[12] Weissman B., Meyer K. (1954). The structure of hyalobiuronic acid and of hyaluronic acid from umbilical cord. J. Am. Chem. Soc..

[13] Itano N., Kimata K. (1996). Expression cloning and molecular characterization of HAS protein, a eukaryotic hyaluronan synthase. J. Biol. Chem..

[14] Itano N., Atsumi F., Sawai T., Yamada Y., Miyaishi O., Senga T., Hamaguchi M., Kimata K. (2002). Abnormal accumulation of hyaluronan matrix diminishes contact inhibition of cell growth and promotes cell migration. Proc. Natl. Acad. Sci. USA.

[15] Scott J.E., Cummings C., Brass A., Chen Y. (1991). Secondary and tertiary structures of hyaluronan in aqueous solution, investigated by rotary shadowing-electron microscopy and computer simulation. Hyaluronan is a very efficient network-forming polymer. Biochem. J..

[16] Salustri A., Yanagishita M., Underhill C.B., Laurent T.C., Hascall V.C. (1992). Localization and synthesis of hyaluronic acid in the cumulus cells and mural granulosa cells of the preovulatory follicle. Dev. Biol..

[17] Tammi R., Tammi M. (1991). Correlations between hyaluronan and epidermal proliferation as studied by [_3_H]glucosamine and [_3_H]thymidine incorporations and staining of hyaluronan on mitotic keratinocytes. Exp. Cell Res..

[18] Horton M.R., Burdick M.D., Strieter R.M., Bao C., Noble P.W. (1998). Regulation of hyaluronan-induced chemokine gene expression by IL-10 and IFN-γ in mouse macrophages. J. Immunol..

[19] D’Agostino A., Stellavato A., Busico T., Papa A., Tirino V., Papaccio G., La Gatta A., De Rosa M., Schiraldi C. (2015). In vitro analysis of the effects on wound healing of high- and low-molecular weight chains of hyaluronan and their hybrid H-HA/L-HA complexes. BMC Cell Biol..

[20] Auvinen P., Tammi R., Parkkinen J., Tammi M., Agren U., Johansson R., Hirvikoski P., Eskelinen M., Kosma V.M. (2000). Hyaluronan in peritumoral stroma and malignant cells associates with breast cancer spreading and predicts survival. Am. J. Pathol..

[21] Setälä L.P., Tammi M.I., Tammi R.H., Eskelinen M.J., Lipponen P.K., Agren U.M., Parkkinen J., Alhava E.M., Kosma V.M. (1999). Hyaluronan expression in gastric cancer cells is associated with local and nodal spread and reduced survival rate. Br. J. Cancer.

[22] Köbel M., Weichert W., Crüwell K., Schmitt W.D., Lautenschläger C., Hauptmann S. (2004). Epithelial hyaluronic acid and CD44v6 are mutually involved in invasion of colorectal adenocarcinomas and linked to patient prognosis. Virchows Arch..

[23] Theocharis A.D., Tsara M.E., Papageorgacopoulou N., Karavias D.D., Theocharis D.A. (2000). Pancreatic carcinoma is characterized by elevated content of hyaluronan and chondroitin sulfate with altered disaccharide composition. Biochim. Biophys. Acta.

[24] Skandalis S.S., Kletsas D., Kyriakopoulou D., Stavropoulos M., Theocharis D.A. (2006). The greatly increased amounts of accumulated versican and decorin with specific post-translational modifications may be closely associated with the malignant phenotype of pancreatic cancer. Biochim. Biophys. Acta.

[25] Cheng X.B., Sato N., Kohi S., Yamaguchi K. (2013). Prognostic impact of hyaluronan and its regulators in pancreatic ductal adenocarcinoma. PLoS ONE.

[26] Abetamann V., Kern HF., Elsässer H.P. (1996). Differential expression of the hyaluronan receptors CD44 and RHAMM in human pancreatic cancer cells. Clin. Cancer Res..

[27] Sugahara K.N., Hirata T., Hayasaka H., Stern R., Murai T., Miyasaka M. (2006). Tumor cells enhance their own CD44 cleavage and motility by generating hyaluronan fragments. J. Biol. Chem..

[28] Kiuchi S., Ikeshita S., Miyatake Y., Kasahara M. (2015). Pancreatic cancer cells express CD44 variant 9 and multidrug resistance protein 1 during mitosis. Exp. Mol. Pathol..

[29] Zhu R., Wang S.C., Sun C., Tao Y., Piao H.L., Wang X.Q., Du M., Da-Jin Li. (2013). Hyaluronan-CD44 interaction promotes growth of decidual stromal cells in human first-trimester pregnancy. PLoS ONE.

[30] Lokeshwar V.B., Mirza S., Jordan A. (2014). Targeting hyaluronic acid family for cancer chemoprevention and therapy. Adv. Cancer Res..

[31] Zhang Y., Wei J., Wang H., Xue X., An Y., Tang D., Yuan Z., Wang F., Wu J., Zhang J. (2012). Epithelial mesenchymal transition correlates with CD24+CD44+ and CD133+ cells in pancreatic cancer. Oncol. Rep..

[32] Wei X., Senanayake T.H., Warren G., Vinogradov S.V. (2013). Hyaluronic acid-based nanogel-drug conjugates with enhanced anticancer activity designed for the targeting of CD44-positive and drug-resistant tumors. Bioconjug. Chem..

[33] Provenzano P.P., Cuevas C., Chang A.E., Goel V.K., Von Hoff D.D., Hingorani S.R. (2012). Enzymatic targeting of the stroma ablates physical barriers to treatment of pancreatic ductal adenocarcinoma. Cancer Cell.

[34] Spicer A.P., McDonald J.A. (1998). Characterization and molecular evolution of a vertebrate hyaluronan synthase gene family. J. Biol. Chem..

[35] Csóka A.B., Scherer S.W., Stern R. (1999). Expression analysis of six paralogous human hyaluronidase genes clustered on chromosomes 3p21 and 7q31. Genomics.

[36] Cheng X.B., Kohi S., Koga A., Hirata K., Sato N. (2016). Hyaluronan stimulates pancreatic cancer cell motility. Oncotarget.

[37] Saarni H., Hopsu-Havu V.K. (1978). The decrease of hyaluronate synthesis by anti-inflammatory steroids in vitro. Br. J. Dermatol..

[38] Goldberg R.L., Toole B.P. (1983). Monensin inhibition of hyaluronate synthesis in rat fibrosarcoma cells. J. Biol. Chem..

[39] Mason R.M., Lineham J.D., Phillipson M.A., Black C.M. (1984). Selective inhibition of proteoglycan and hyaluronate synthesis in chondrocyte cultures by cyclofenil diphenol, a non-steroidal weak oestrogen. Biochem. J..

[40] Prehm P. (1985). Inhibition of hyaluronate synthesis. Biochem. J..

[41] Gressner A.M., Haarmann R. (1988). Effect of n-butyrate on the synthesis of sulfated glycosaminoglycans and hyaluronate by rat liver fat-storing cells (Ito cells). Biochem. Pharmacol..

[42] Smith T.J. (1988). Glucocorticoid regulation of glycosaminoglycan synthesis in cultured human skin fibroblasts: Evidence for a receptor-mediated mechanism involving effects on specific de novo protein synthesis. Metabolism.

[43] Smith T.J. (1990). Retinoic acid inhibition of hyaluronate synthesis in cultured human skin fibroblasts. J. Clin. Endocrinol. Metab..

[44] Gressner A.M. (1991). Proliferation and transformation of cultured liver fat-storing cells (perisinusoidal lipocytes) under conditions of β-d-xyloside-induced abrogation of proteoglycan synthesis. Exp. Mol. Pathol..

[45] Honda A., Noguchi N., Takehara H., Ohashi Y., Asuwa N., Mori Y. (1991). Cooperative enhancement of hyaluronic acid synthesis by combined use of IGF-I and EGF, and inhibition by tyrosine kinase inhibitor genistein, in cultured mesothelial cells from rabbit pericardial cavity. J. Cell Sci..

[46] August E.M., Duncan K.L., Malinowski N.M., Cysyk R.L. (1993). Inhibition of fibroblast hyaluronic acid production by suramin. Oncol. Res..

[47] Zaharevitz D.W., Chisena C.A., Duncan K.L., August E.M., Cysyk R.L. (1993). Vanadate inhibition of hyaluronic acid synthesis in Swiss 3T3 fibroblasts. Biochem. Mol. Biol. Int..

[48] Nakamura T., Takagaki K., Shibata S., Tanaka K., Higuchi T., Endo M. (1995). Hyaluronic-acid-deficient extracellular matrix induced by addition of 4-methylumbelliferone to the medium of cultured human skin fibroblasts. Biochem. Biophys. Res. Commun..

[49] Yaron I., Shirazi I., Judovich R., Levartovsky D., Caspi D., Yaron M. (1999). Fluoxetine and amitriptyline inhibit nitric oxide, prostaglandin E2, and hyaluronic acid production in human synovial cells and synovial tissue cultures. Arthritis Rheum..

[50] Ueki N., Taguchi T., Takahashi M., Adachi M., Ohkawa T., Amuro Y., Hada T., Higashino K. (2000). Inhibition of hyaluronan synthesis by vesnarinone in cultured human myofibroblasts. Biochim. Biophys. Acta.

[51] Jokela T.A., Jauhiainen M., Auriola S., Kauhanen M., Tiihonen R., Tammi M.I., Tammi R.H. (2008). Mannose inhibits hyaluronan synthesis by down-regulation of the cellular pool of UDP-*N*-acetylhexosamines. J. Biol. Chem..

[52] Kultti A., Kärnä R., Rilla K., Nurminen P., Koli E., Makkonen K.M., Si J., Tammi M.I., Tammi R.H. (2010). Methyl-β-cyclodextrin suppresses hyaluronan synthesis by down-regulation of hyaluronan synthase 2 through inhibition of Akt. J. Biol. Chem..

[53] Freudenberger T., Röck K., Dai G., Dorn S., Mayer P., Heim H.K., Fischer J.W. (2011). Estradiol inhibits hyaluronic acid synthase 1 expression in human vascular smooth muscle cells. Basic Res. Cardiol..

[54] Röck K., Grandoch M., Majora M., Krutmann J., Fischer J.W. (2011). Collagen fragments inhibit hyaluronan synthesis in skin fibroblasts in response to ultraviolet B (UVB): New insights into mechanisms of matrix remodeling. J. Biol. Chem..

[55] Evans R.R., Relling M.V. (1992). Automated high-performance liquid chromatographic assay for the determination of 7-ethoxycoumarin and umbelliferone. J. Chromatogr..

[56] Kudo D., Kon A., Yoshihara S., Kakizaki I., Sasaki M., Endo M., Takagaki K. (2004). Effect of a hyaluronan synthase suppressor, 4-methylumbelliferone, on B16F-10 melanoma cell adhesion and locomotion. Biochem. Biophys. Res. Commun..

[57] Yoshihara S., Kon A., Kudo D., Nakazawa H., Kakizaki I., Sasaki M., Endo M., Takagaki K. (2005). A hyaluronan synthase suppressor, 4-methylumbelliferone, inhibits liver metastasis of melanoma cells. FEBS Lett..

[58] Nakazawa H., Yoshihara S., Kudo D., Morohashi H., Kakizaki I., Kon A., Takagaki K., Sasaki M. (2006). 4-methylumbelliferone, a hyaluronan synthase suppressor, enhances the anticancer activity of gemcitabine in human pancreatic cancer cells. Cancer Chemother. Pharmacol..

[59] Lokeshwar V.B., Lopez L.E., Munoz D., Chi A., Shirodkar S.P., Lokeshwar S.D., Escudero D.O., Dhir N., Altman N. (2010). Antitumor activity of hyaluronic acid synthesis inhibitor 4-methylumbelliferone in prostate cancer cells. Cancer Res..

[60] Twarock S., Tammi M.I., Savani R.C., Fischer J.W. (2010). Hyaluronan stabilizes focal adhesions, filopodia, and the proliferative phenotype in esophageal squamous carcinoma cells. J. Biol. Chem..

[61] Wang T.P., Pan Y.R., Fu C.Y., Chang H.Y. (2010). Down-regulation of UDP-glucose dehydrogenase affects glycosaminoglycans synthesis and motility in HCT-8 colorectal carcinoma cells. Exp. Cell Res..

[62] Urakawa H., Nishida Y., Wasa J., Arai E., Zhuo L., Kimata K., Kozawa E., Futamura N., Ishiguro N. (2012). Inhibition of hyaluronan synthesis in breast cancer cells by 4-methylumbelliferone suppresses tumorigenicity in vitro and metastatic lesions of bone in vivo. Int. J. Cancer.

[63] Piccioni F., Malvicini M., Garcia M.G., Rodriguez A., Atorrasagasti C., Kippes N., Piedra Buena I.T., Rizzo M.M., Bayo J., Aquino J. (2012). Antitumor effects of hyaluronic acid inhibitor 4-methylumbelliferone in an orthotopic hepatocellular carcinoma model in mice. Glycobiology.

[64] Arai E., Nishida Y., Wasa J., Urakawa H., Zhuo L., Kimata K., Kozawa E., Futamura N., Ishiguro N. (2011). Inhibition of hyaluronan retention by 4-methylumbelliferone suppresses osteosarcoma cells in vitro and lung metastasis in vivo. Br. J. Cancer.

[65] Lompardía S.L., Papademetrio D.L., Mascaró M., Álvarez E.M., Hajos S.E. (2013). Human leukemic cell lines synthesize hyaluronan to avoid senescence and resist chemotherapy. Glycobiology.

[66] Kakizaki I., Kojima K., Takagaki K., Endo M., Kannagi R., Ito M., Maruo Y., Sato H., Yasuda T., Mita S. (2004). A novel mechanism for the inhibition of hyaluronan biosynthesis by 4-methylumbelliferone. J. Biol. Chem..

[67] Kultti A., Pasonen-Seppänen S., Jauhiainen M., Rilla K.J., Kärnä R., Pyöriä E., Tammi R.H., Tammi M.I. (2009). 4-Methylumbelliferone inhibits hyaluronan synthesis by depletion of cellular UDP-glucuronic acid and downregulation of hyaluronan synthase 2 and 3. Exp. Cell Res..

[68] Nagase H., Kudo D., Suto A., Yoshida E., Suto S., Negishi M., Kakizaki I., Hakamada K. (2016). 4-Methylumbelliferone suppresses hyaluronan synthesis and tumor progression in scid mice intra-abdominally inoculated with pancreatic cancer cells. Pancreas.

[69] Takeda S., Aburada M. (1981). The choleretic mechanism of coumarin compounds and phenolic compounds. J. Pharmacobiodyn..

[70] Abate A., Dimartino V., Spina P., Costa P.L., Lombardo C., Santini A., Del Piano M., Alimonti P. (2001). Hymecromone in the treatment of motor disorders of the bile ducts: A multicenter, double-blind, placebo-controlled clinical study. Drugs Exp. Clin. Res..

[71] Hingorani S.R., Harris W.P., Beck J.T., Berdov B.A., Wagner S.A., Pshevlotsky E.M., Tjulandin S.A., Gladkov O.A., Holcombe R.F., Korn R. (2016). Phase Ib study of PEGylated recombinant human hyaluronidase and gemcitabine in patients with advanced pancreatic cancer. Clin. Cancer Res..

[72] Thompson C.B., Shepard H.M., O’Connor P.M., Kadhim S., Jiang P., Osgood R.J., Bookbinder L.H., Li X., Sugarman B.J., Connor R.J. (2010). Enzymatic depletion of tumor hyaluronan induces antitumor responses in preclinical animal models. Mol. Cancer Ther..

[73] Yoshida E., Kudo D., Nagase H., Shimoda H., Suto S., Negishi M., Kakizaki I., Endo M., Hakamada K. (2016). Antitumor effects of the hyaluronan inhibitor 4-methylumbelliferone on pancreatic cancer. Oncol. Lett..

[74] Miyazawa M., Katsuda M., Maguchi H., Katanuma A., Ishii H., Ozaka M., Yamao K., Imaoka H., Kawai M., Hirono S. (2016). Phase II clinical trial using novel peptide cocktail vaccine as a postoperative adjuvant treatment for surgically resected pancreatic cancer patients. Int. J. Cancer.

[75] Lei J., Wu Z., Jiang Z., Li J., Zong L., Chen X., Duan W., Xu Q., Zhang L., Han L. (2016). Pancreatic carcinoma-specific immunotherapy using novel tumor specific cytotoxic T cells. Oncotarget.

[76] Mace T.A., Shakya R., Pitarresi J.R., Swanson B., McQuinn C.W., Loftus S., Nordquist E., Cruz-Monserrate Z., Yu L., Young G. (2016). IL-6 and PD-L1 antibody blockade combination therapy reduces tumour progression in murine models of pancreatic cancer. Gut.

[77] Tewari N., Zaitoun A.M., Arora A., Madhusudan S., Ilyas M., Lobo D.N. (2013). The presence of tumour-associated lymphocytes confers a good prognosis in pancreatic ductal adenocarcinoma: An immunohistochemical study of tissue microarrays. BMC Cancer.

[78] Homma Y., Taniguchi K., Murakami T., Nakagawa K., Nakazawa M., Matsuyama R., Mori R., Takeda K., Ueda M., Ichikawa Y. (2014). Immunological impact of neoadjuvant chemoradiotherapy in patients with borderline resectable pancreatic ductal adenocarcinoma. Ann. Surg. Oncol..

[79] Maeda T., Nagano S., Ichise H., Kataoka K., Yamada D., Ogawa S., Koseki H., Kitawaki T., Kadowaki N., Takaori-Kondo A. (2016). Regeneration of CD8αβ T Cells from T-cell-derived iPSC imparts potent tumor antigen-specific cytotoxicity. Cancer Res..

[80] McKallip R.J., Ban H., Uchakina O.N. (2015). Treatment with the hyaluronic Acid synthesis inhibitor 4-methylumbelliferone suppresses LPS-induced lung inflammation. Inflammation.

[81] Colombaro V., Declèves A.E., Jadot I., Voisin V., Giordano L., Habsch I., Nonclercq D., Flamion B., Caron N. (2013). Inhibition of hyaluronan is protective against renal ischaemia-reperfusion injury. Nephrol. Dial. Transplant..

[82] Yoshioka Y., Kozawa E., Urakawa H., Arai E., Futamura N., Zhuo L., Kimata K., Ishiguro N., Nishida Y. (2013). Suppression of hyaluronan synthesis alleviates inflammatory responses in murine arthritis and in human rheumatoid synovial fibroblasts. Arthritis Rheum..

